# Strategic nucleic acid detection approaches for diagnosing African swine fever (ASF): navigating disease dynamics

**DOI:** 10.1186/s13567-024-01386-8

**Published:** 2024-10-07

**Authors:** Yuanshou Zhu, Meng Zhang, Zhijun Jie, Shujuan Guo, Zhigang Zhu, Sheng-ce Tao

**Affiliations:** 1 School of Health Science and Engineering, University of Shanghai for Science and Technology, Shanghai, 200093 China; 2 Shanghai Center for Systems Biomedicine, Key Laboratory of Systems Biomedicine (Ministry of Education), Shanghai Jiao Tong University, Shanghai, 200240 China; 3 Department of Pulmonary and Critical Care Medicine, Shanghai Fifth People’s Hospital, Fudan University, Shanghai, 200240 China; 4 Center of Community-Based Health Research, Fudan University, Shanghai, 200240 China

**Keywords:** ASF, ASFV variants, ASF live attenuated vaccines, CRISPR-based diagnostic, disease dynamics and detection strategies

## Abstract

African swine fever (ASF) is a devastating disease caused by African swine fever virus (ASFV) and leads to significant economic losses in the pig farming industry. Given the absence of an effective vaccine or treatment, the mortality rate of ASF is alarmingly close to 100%. Consequently, the ability to rapidly and accurately detect ASFV on site and promptly identify infected pigs is critical for controlling the spread of this pandemic. The dynamics of the ASF virus load and antibody response necessitate the adoption of various detection strategies at different stages of infection, a topic that has received limited attention to date. This review offers detailed guidance for choosing appropriate ASF diagnostic techniques tailored to the clinical manifestations observed from the acute to chronic phases, including asymptomatic cases. We comprehensively summarize and evaluate the latest advancements in ASFV detection methods, such as CRISPR-based diagnostics, biosensors, and microfluidics. Additionally, we address the challenges of false negatives or positives due to ASF variants or the use of injected live attenuated vaccines. This review provides an exhaustive list of diagnostic tests suitable for detecting each stage of symptoms and potential target genes for developing new detection methods. In conclusion, we highlight the current challenges and future directions in ASFV detection, underscoring the need for continued research and innovation in this field.

## Introduction

African swine fever (ASF) is a highly infectious and deadly hemorrhagic disease affecting both domestic and wild pigs. Owing to its significant impact, the World Organization for Animal Health (WOAH, previously known as OIE) has classified it as a “notifiable disease” [1, 2]. Typically, ASF symptoms include high fever, skin cyanosis, and acute hemorrhages in the lymph nodes, making it difficult to distinguish it from classical swine fever (CSF) through clinical or postmortem examinations [3, 4]. Since its initial identification in Kenya in 1921, African swine fever virus (ASFV) has expanded its reach from Africa to Europe, the Americas, and Asia on multiple occasions [5, 6]. The disease first appeared in China, the world’s leading pig producer, in August 2018, resulting in 165 outbreaks across 32 provinces and approximately 1 193 000 pig fatalities within just two years [7]. The genetic and antigenic diversity of ASFV, coupled with inadequate cross-protective immunity, complicates the development of effective vaccines and treatments [8, 9]. Thus, immediate, precise, and onsite detection of ASFV, followed by swift culling of infected pigs, is essential for outbreak management. Diagnosis typically involves detecting both the viral genome and antibodies [10, 11], with serological tests offering rapid results but failing to identify subacute or early-stage infections owing to delayed antibody responses post infection. Nucleic acid testing (NAT), while highly sensitive and specific, requires skilled operators and advanced equipment. The challenge lies in selecting appropriate detection strategies to match the varying virus loads and antibody dynamics associated with ASF [12].

**Figure 1:**
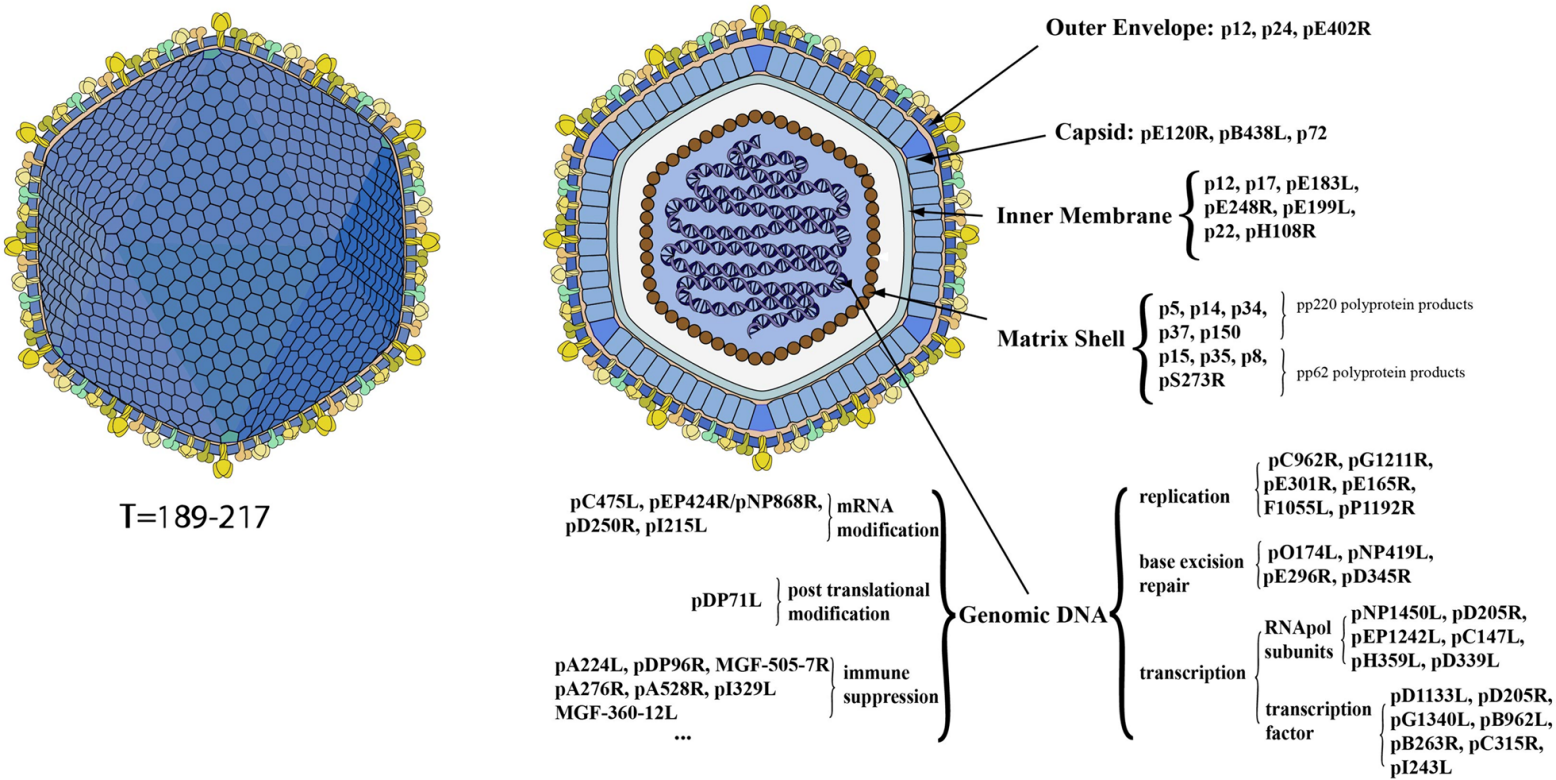
**Illustration of the ASFV structure and genome.** ASFV belongs to the family *Asfarviridae* and has an icosahedral enveloped virion. From the outside to the inside are the outer envelope, capsid, inner membrane, matrix shell, and genome. The genome of ASFV is dsDNA and encodes more than 150 ORFs of ∼50 structural proteins and 100 immunoregulatory proteins. Most structural proteins, such as pp62, pp220, p72, p30, and CD2v, are major components of ASF virus particles and play important roles in the process of virus attachment, entry, and replication. Other ASFV-encoded proteins, such as pC475, pDP71L, pA224, pO174L, and pNP1450L, are involved in mRNA modification, posttranslational modification, immune suppression, base excision repair, and transcription [131].

ASFV, a double-stranded DNA (dsDNA) arbovirus, features an icosahedral envelope and belongs to the *Asfarviridae* family [13, 14]. Its expansive genome spans 170–194 kb and comprises a central conserved region (C region, ~ 125 kb), a left variable region (LVR, 38–48 kb), and a right variable region (RVR, 13–22 kb), encoding more than 150 open reading frames (ORFs) for approximately 50 structural proteins and 100 immunoregulatory proteins induced by the host [15–17]. As shown in Figure 1, key structural proteins, such as pp62, pp220, p72, p30, and CD2v, are essential for virus attachment, entry, and replication [18–20]. The virus has been categorized into 24 genotypes based on the p72 protein encoded by the B646L gene and into 8 serogroups according to the CD2v protein from the EP402R gene [21]. Although p72 genotyping is widely accepted, it sometimes lacks the resolution necessary to distinguish viruses with varying biological phenotypes. Therefore, additional gene regions, such as p54, B602L, and 9GL, have been employed for more detailed genetic analyses [22–24]. The p54 protein, encoded by the E183L gene, improves intragenotypic resolution, whereas the central variable region (CVR) of the B602L gene aids in subtype differentiation. Various genotyping methods have been developed to assess evolutionary trends, employing additional gene regions and intergenic sequences for enhanced discrimination [25–30]. Genotyping during outbreaks is crucial for tracing virus origins and distinguishing between closely related strains. The ongoing emergence of new variants further complicates ASF diagnosis and treatment [31], necessitating differentiation from other major swine viruses, such as classical swine fever virus (CSFV), pseudorabies virus (PRV), porcine parvovirus (PPV), and porcine circovirus 2 (PCV2) [32]. Thus, a detection method that is portable, accurate, and rapid is essential for effective ASFV monitoring.

This review begins by outlining the structure, genetic makeup, and various genotyping methods of ASFV. This paper then comprehensively reviews current detection techniques, highlighting the advantages and disadvantages of each technique, with a special focus on emerging clustered regularly interspaced short palindromic repeat (CRISPR)-based systems and biosensors. We also detail the complete workflow of ASFV NAT methods and compile extensive lists of reported genes and gene mutations detected in potential vaccine candidates or naturally less virulent strains, which could serve as targets for ASF detection. Additionally, we discuss the occurrence of false negatives or positives due to ASFV variants or live attenuated vaccines, especially vaccines based on the deletion of virulence-associated genes (e.g., ASFV-Kenya-IX-1033-∆CD2v, ASFV-ΔQP509L/QP383R and ASFV-G-ΔA137R strains) [8, 33–35]. Considering the disease’s dynamics, we offer advice on selecting diagnostic techniques based on different clinical presentations, from the acute to chronic stages, including asymptomatic infections. Finally, we identified the main challenges and future directions in ASFV detection, emphasizing that applying the most suitable detection methods, combining antibody and nucleic acid tests, will increase ASF control efforts.

## Outline of the ASFV nucleic acid testing workflow

**Figure 2:**
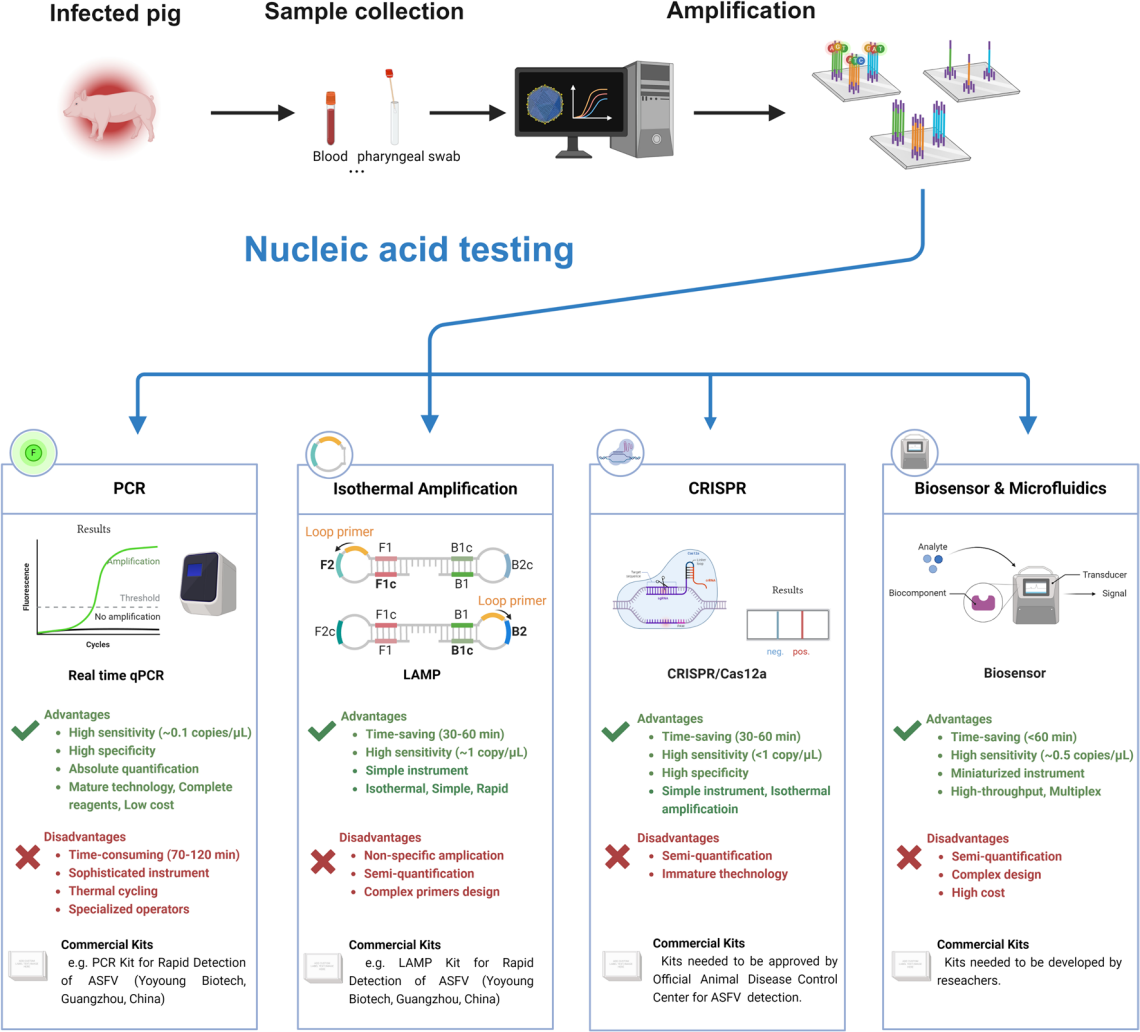
**Workflow of ASFV nucleic acid testing.** The whole detection process consists of sample collection, amplification, and nucleic acid testing. The samples were collected from the blood or pharyngeal swabs of infected pigs. Sample treatment includes DNA extraction from deactivated viruses or simple lysis by specialized reagents. Various nucleic acid testing methods, including PCR, isothermal amplification, CRISPR, and biosensors and microfluidics, can be used for ASFV detection. The advantages and disadvantages of different methods are also listed.

The diagnosis of ASF involves three steps: sample preparation, amplification, and nucleic acid testing (Figure 2).

For sample collection, specimens are usually obtained from the blood or pharyngeal swabs of infected pigs. Then, samples need to be processed by DNA extraction from deactivated viruses or simple lysis by chemical reagents. For example, Ceruti et al*.* tested two extraction methods, a standardized silica-based DNA extraction kit and simple nonpurification DNA isolation (QIAGEN ATL lysis buffer and heating at 70 °C for 20 min), and reported that both methods can meet the demands of ASFV detection [36]. Nishi et al. also reported a highly sensitive and rapid method for the simultaneous detection of ASFV and CSFV using a simplified nucleic acid extraction technique [37]. In terms of DNA extraction, solid-phase extraction (SPE) has been widely used in clinical detection with high accuracy and relies on silica membranes, beads, filter papers, or polymer resins. In addition, magnetic bead-based methods, anion-exchange resins, and guanidinium thiocyanate-phenol‒chloroform extractions are also commonly applied for nucleic acid extraction [38]. DNA extraction can provide pure nucleic acids but can cause some loss of DNA. In contrast, direct amplification can reduce DNA loss and operation time but may also cause nucleic acid impurities, inhibit the reaction, and reduce sensitivity.

For amplification and NAT, different detection methods have their own advantages and disadvantages, which are also listed in Figure 2. A variety of NAT methods, such as polymerase chain reaction (PCR), isothermal amplification, CRISPR, biosensors and microfluidics, have the capacity to detect ASFV. This review comprehensively introduces and evaluates these methods in detail below.

## Sequencing technology

Whole-genome sequencing (WGS) is a fast and affordable way to obtain detailed information about unknown pathogens based on high-throughput sequencing (HTS). Given that the genome of ASFV is very complex and that complete genetic information is currently unavailable, it is important to utilize WGS to efficiently obtain viral genome sequences for genomic and epidemiological studies.

The popular WGS methods currently used can be generally classified as short-read sequencing (SRS) [39] or long-read sequencing (LRS) [40]. SRS can produce many high-precision sequencing reads for transcriptomic profiling via Illumina sequencing. Using SRS-based RNA-seq, Jaing et al*.* conducted an analysis to characterize differentially expressed genes in pigs infected with a low-pathogenicity ASFV [41]. Approximately 60% of the ORFs (109 genes) were detected in the circulating monocytes of highly pathogenic Georgia 2007/1-infected pigs. However, owing to the limitations in coping with complex and large transcriptomes, comprehensive annotation of transcriptomes is inefficient, which gives rise to the third-generation LRS. Currently, the Oxford Nanopore MinION rules the LRS market and has been proven to be a valuable tool for tracking ASFV because of its ability to produce long reads in real time. O’Donnell et al*.* carefully investigated the capacity of the MinION sequence-sensing device to act as an ASF fast analysis sequencing tool (ASF-FAST) [42]. To further improve the efficiency and universality of these methods, SRS and LRS can be combined to trace ASFV. Torma et al*.* coupled the Illumina SRS and Oxford Nanopore Technologies LRS platforms with multiple library preparation methods (amplified and direct cDNA sequencing and native RNA sequencing) to construct the ASFV transcriptomic atlas [43]. Similarly, Spinard et al*.* assembled a contig representing the full-length genome (183 687 base pairs) into a single contig using both Illumina and Nanopore sequencing and reported that DR-1980 belongs to the genotype I ASFV [44].

Obviously, HTS adapts to the development needs of the era of big data. In addition to pathogen identification, HTS can also be applied for antibiotic resistance detection, human and plant genome sequencing, environmental microorganism analysis, and food safety monitoring. Unfortunately, HTS still has several shortcomings, including a high detection cost, long reaction time and highly specialized data analysis. Different technologies have their own usage scenarios, so HTS is commonly used for unknown pathogen identification and new mutation/variant analysis instead of routine nucleic acid detection.

## Polymerase chain reaction (PCR)

Real-time quantitative PCR (RT–qPCR) is currently recommended as the gold standard for the detection of ASFV and continues to evolve. With specifically designed probes labelled with fluorophores, RT‒qPCR has increased sensitivity and ability to quantify fluorescence signals. Because of its high degree of conservation, the B646L gene is the most widely used target for ASFV detection. In different application scenarios, various methods targeting the B646L gene have been developed.

By utilizing lyophilized powder reagents, Wang et al*.* exploited a novel RT‒qPCR method to diagnose ASF in domestic pigs with high repeatability, low time consumption and cost. Without cross‐reactivity with other critical swine pathogens, this method displayed tenfold greater sensitivity than the qPCR assay recommended by WOAH [45]. Similarly, Hwang et al*.* presented a fast RT‒qPCR assay based on the B646L gene to identify 24 genotypes of ASFV [46]. The limit of detection (LOD) for the most virulent genotype was 6.91 copies/reaction. Moreover, under certain circumstances, such as in water, which is an important transmission vector, a low concentration of ASFV poses a challenge for detection. To accurately detect ASFV in water, Wu et al*.* combined qPCR with a rapid and efficient water-borne virus enrichment system modified with ferric hydroxide colloids [47]. After the spiked water was concentrated from 10 L to 4 mL, the LOD decreased to 1 × 10^–1.11^ GU/mL (genomic units per milliliter) from 1 × 10^2.71^ GU/mL.

To increase the efficiency and accuracy of PCR, multiplex PCR (mPCR) was developed to detect multiple targets simultaneously in a single reaction. For example, Qian et al*.* established an mPCR method to distinguish genotype I and II ASFV by targeting the B646L, F1055L, and E183L genes [48]. In addition to multigene detection of one swine virus, several mPCR methods exist for the simultaneous detection of multiple swine viruses. Chen et al*.* designed three pairs of PCR primers and TaqMan probes targeting the ASFV B646L gene, CSFV 5′ untranslated region, and porcine reproductive and respiratory syndrome virus (PRRSV) ORF7 gene in a single reaction [49]. Similarly, Liu et al*.* developed a one-step triplex RT‒qPCR method to identify ASFV, CSFV, and atypical porcine pestivirus (APPV) with an LOD of 25.2 copies/μL [50]. The virotype ASFV 2.0 PCR Kit (Indical Bioscience, USA), a triplex real-time PCR, was also used for rapid and highly reliable detection of ASFV within 60 min [51]. In general, RT‒qPCR has played a significant role in the detection of ASFV. Nevertheless, RT‒qPCR also has several drawbacks, including expensive thermal cycling instruments, professional operators, and a high reliance on high-quality samples. Fortunately, several limitations have been overcome by the emergence of isothermal amplification, microfluidics, and CRISPR technology.

## Isothermal amplification

Although PCR has been extensively applied for the detection of ASFV, it is typically limited by the use of sophisticated thermocyclers, skilled professionals and relatively long reaction times. Moreover, the collected samples need to be transported to the central laboratory for PCR, which not only delays ASFV identification but also poses a biosafety risk. In view of this, PCR is not suitable for onsite detection of ASFV in resource-constrained settings. Because of the lower and constant reaction temperature and minimal power consumption, several isothermal amplification methods, including loop-mediated isothermal amplification (LAMP) [52], recombinase polymerase amplification (RPA) [36], rolling circle amplification (RCA) [53], and hybrid chain reaction (HCR) [54], have been developed for ASFV detection. They have great potential for point-of-care testing (POCT) when combined with signal readout technology, especially for LAMP and RPA.

### Loop-mediated isothermal amplification (LAMP)

LAMP, which relies on strand displacement DNA polymerase (Bst) and four primers (inner and outer primers), can achieve 10^9^ ~ 10^10^-fold amplification within 15 ~ 60 min at a constant temperature of 60 ~ 65 °C [55]. Over the past few years, a series of LAMP-based ASFV detection methods have been established. Among these methods, the greatest difference lies in terminal signal detection methods, including electrophoresis, turbidimetry, colorimetry, and some acid–base indicators [56]. For example, Wang et al*.* developed a real-time visual LAMP assay based on EvaGreen for the detection of the p10 gene of ASFV [57]. In comparison, Zhang’s group selected neutral red, a pH-sensitive dye, as a color shift indicator to establish a visual LAMP assay [58]. During LAMP detection of the ASFV p72 gene, the neutral red color sharply changed from orange (negative) to pink (positive). Using both fluorometric and colorimetric ASFV-LAMP methods, Bohorquez et al*.* detected ASFV plasmids at concentrations as low as 1 copy/μL [59]. In addition to the use of a fluorescent dye, Wang et al*.* developed a cleaved probe-based LAMP (CP-LAMP) detection method to target the ASFV 9GL gene [60]. The enzyme activity of ribonuclease H2 (RNase H2) can be activated only when the probe is perfectly matched with the template, resulting in hydrolytic release of the fluorophore.

To explore novel specific-signal-output strategies in isothermal amplification, He et al*.* designed a visual-signal-report system to filter and magnify the target in LAMP, which is composed of two processes: LAMP with specific signal filtration and self-replication catalyzed hairpin assembly (SRCHA) for specific signal magnification and output [61]. Another sensitive nanomaterial, carbon nanodots (CNDs), was also combined with LAMP for ultrasensitive detection of ASFV [62]. CNDs are spherical fluorescent materials less than 10 nm in diameter that have good water solubility and biocompatibility, stable chemical properties, adjustable photoluminescence, and low toxicity [63]. Some CNDs respond significantly to pH changes and serve as pH indicators. In contrast, surface plasmon resonance (SPR) is a real-time, label-free and simple-to-operate detection method that can measure refractive index changes on the surface of a sensor chip due to biological interactions caused by optical principles. Zhang et al*.* used fluorescence and SPR methods for real-time detection of LAMP products of ASFV [64]. In recent years, with the discovery of its trans-cleavage activity, CRISPR has become a popular diagnostic tool for nucleic acids. Qian et al*.* devised a one-pot detection method that adopted filter paper to extract nucleic acids from swine blood and utilized the CRISPR/Cas12a-based LAMP reaction to detect ASFV [65]. To cope with these conditions, several researchers have developed direct colorimetric LAMP assays for the rapid detection of ASFV without DNA extraction [66, 67].

In summary, LAMP is currently one of the most common isothermal amplification methods; it involves various terminal signal detection methods and can be easily integrated with diverse technologies. For fieldable detection of ASFV, LAMP is a promising choice because of its short reaction time, simple operation, low amplification temperature and electricity consumption. However, LAMP is a single-channel detection method, and it can hardly achieve simultaneous detection of a large number of samples. Because of the greater number of primers used in LAMP, nonspecific amplification and cross-reaction are still concerns, which largely limits multiplex detection within one tube. To realize multichannel LAMP detection, electronic chip technology will become a future development direction. Aerosol contamination can be avoided by one-pot and fully enclosed LAMP detection. A miniaturized device will pave the way for onsite detection of ASFV.

### Recombinase polymerase amplification (RPA)

Compared with LAMP, RPA has a lower reaction temperature (37 ~ 42 °C) and can achieve ~ 10^12^-fold amplification within 20 ~ 40 min [68]. In recent years, many researchers have used RPA for ASFV detection. For example, Ilya et al*.* developed a conventional real-time RPA assay (exo-probe-based) that targets the CP204L gene to identify ASFV genotypes I and II [69]. Fan et al. devised a clinical validation of both RPA and RAA (recombinase-aided amplification) targeting the B646L gene (p72) for rapid detection of ASFV [70]. Because of the high cost of probes, Zhang et al*.* constructed a directly visualized SYBR Green I-staining RPA (RPAS) method to detect ASFV with an LOD of 10^3^ copies/μL [71]. To further amplify the signal output, a stem‒loop DNA probe-based reporter system assisted by exonuclease III (Exo III) cleavage was established by Zhao’s group [72]. This platform uses fluorescent or biotin-labelled DNA probes to enable visual detection of ASFV via the naked eye at an LOD as low as 2 copies/μL. Another technique, the lateral flow dipstick (LFD)/strip (LFS), is widely used in the field of diagnosis and has excellent specificity and simplicity. Many researchers have combined RPA with LFD/LFS to develop rapid ASFV detection methods [73–76]. The key of RPA-LFD/LFS is the production of DNA duplexes with a single-stranded tail, which can hybridize with gold nanoparticle (AuNP)-labelled oligonucleotide detection probes.

To increase the sensitivity of LFD/LFS, Wen et al*.* coupled quantum dot microspheres (QDMs) [77] with a FITC polyclonal antibody (QDM-FITC PcAb) to establish a quantitative QDM-based RAA-LFS method and overcome the instrument constraints in ASF diagnosis [78]. This method has a detection limit of 1 copy for ASFV plasmids containing the B646L gene and 100 copies/g for DNA extracts from clinical samples with a short detection time of less than 25 min. To simplify the operation process, nonpurification DNA isolation (lysis buffer and heating, 70 °C for 20 min) is applied in ASFV-RPA to achieve onsite detection [36, 59, 79]. To distinguish ASFV from CSFV, Wu et al*.* developed a double RAA gel electrophoresis detection method with an RAA nucleic acid test strip [80]. Since the emergence of CRISPR, an increasing number of CRISPR-RPA/RAA methods have been established for ASFV detection because of their similar reaction temperatures [81, 82]. The CRISPR system is considered an ideal strategy for specifically detecting amplification products and amplifying signals.

However, because the RPA reaction system is complex and involves at least three main enzymes and other accessory proteins, direct integration with other technologies to develop one-pot reactions is challenging. The general approach is to separate upstream RPA amplification and downstream detection technologies, which can easily cause aerosol contamination and cross reactions. Under these circumstances, specially designed tubes or microfluidic chips are devised to achieve physical isolation and mixing. Unlike for LAMP, for RPA, there is no primer design website or tool available, which increases the difficulty and randomness of primer screening. Moreover, when the length of the primers is relatively long, primer dimers and stem‒loop structures can easily form, resulting in nonspecific amplification. However, even so, RPA plays an increasingly important role in POCT scenes because of its lower reaction temperature, lyophilized pellet format, and high convenience.

## CRISPR-based diagnostic methods

In recent years, CRISPR technology has become the latest and most promising technique in the field of diagnostics. Many Cas proteins (Cas9, Cas12, Cas13, Cas14, etc.) have been utilized in combination with technologies such as signal amplification and fluorescent, colorimetric, potentiometric, and LFD/LFS detection to develop highly sensitive and specific diagnostic tools [83, 84]. In 2020, the CRISPR pioneers Jennifer Doudna and Emmanuelle Charpentier won the Nobel prize in chemistry because of their breakthrough research on CRISPR, which paved the way for gene editing and further emergence of the whole new field of CRISPR diagnostics [85].

The core of CRISPR-based detection technology is the ability to identify the target specifically and significantly amplify the signal. Current CRISPR-based ASFV detection methods include sample amplification (PCR/LAMP/RPA), CRISPR detection (Cas12a/Cas13a), and LFD/LFS readout. For example, Cas13a was utilized with RPA/RAA and LFA to develop supersensitive diagnostic methods for ASF by Ren et al*.* [86] and Wei et al*.* [87]. Similarly, Cas12a was utilized with PCR/RPA and LFA to develop fieldable ASFV detection methods by Wu et al*.* [88] and Lu et al*.* [89]. Nevertheless, separating amplification and detection can easily cause aerosol contamination, which prompts researchers to invent a one-pot and fully sealed visual method for ASFV detection [90, 91]. The LAMP/RPA reagents were incubated at the bottom of the tube, while the Cas12a reagents were added within the lid. When the amplification is complete, the Cas12a reagents are centrifuged into the bottom for product cleavage. In comparison, Hu et al*.* creatively designed a tube-in-tube vessel structure consisting of an inner tube and an outer tube to physically separate Cas13a from the RPA mixture, which was verified by detecting the DNA virus ASFV [92]. To avoid nucleic acid extraction, Cao et al*.* coupled direct PCR with Cas12a and an LFA to detect ASFV at an LOD of 4 copies/μL [93].

To amplify the terminal signal of CRISPR, three novel strategies were proposed for ASFV detection. First, Mao et al*.* reported an advanced system integrating RPA, Cas12a, and magnetic bead (MB)-ssDNA-ALP (alkaline phosphatase) for dual-mode analysis of ASFV genes, including colorimetry and fluorescence [94]. The ALP released by Cas12a can catalyze the hydrolysis of para-nitrophenyl phosphate (pNPP) to para-nitrophenol (pNP), which results in a significant change in color from colorless to yellow. Second, Wang et al*.* combined surface-enhanced Raman scattering (SERS) with the Cas12a system for amplification-free gene detection of ASFV [95]. A SERS-sensing probe was constructed by conjugating plasmonic SERS tags on the MB surface with ssDNA as a linker. Third, Wang et al*.* proposed the use of a CRISPR/Cas9-mediated lateral flow nucleic acid assay (CASLFA) for ASFV detection [96]. Two different AuNP‒DNA probes were utilized for two detection routes: the DNA unwinding-based hybridization route and the sgRNA anchoring-based hybridization route. Furthermore, some researchers have also employed the trimeric G-quadruplex structure in the Cas12a system for the diagnosis of ASFV [97, 98].

To realize high-throughput and multiplex detection, He et al*.* combined Cas12a with a disposable cartridge to develop an all-solution phase and isothermal detection system for ASFV [99]. Tian et al*.* devised a handheld device to exploit the orthogonal cleavage activity of the Cas12a and Cas13a systems for dual-gene detection of ASFV and SARS-CoV-2 [100]. In addition to the use of orthogonal Cas proteins, Zeng et al*.* also harnessed multiplex crRNA in one Cas12a system to achieve amplification-free ASFV diagnosis [101]. The LOD reached ∼1 pM, which was ∼64 times greater than that of the single-crRNA system.

In summary, CRISPR-based diagnostic methods appear to be highly sensitive, specific, and capable of one-step detection of ASFV. Different Cas proteins have their own properties and functions; therefore, predicting and identifying new CRISPR systems are currently the hottest topics in biology. In 2023, Zhang’s group identified 188 previously unreported CRISPR gene modules and characterized a candidate type VII system [102]. However, numerous obstacles may still hamper the widespread application of CRISPR, including protospacer adjacent motif (PAM) sequence dependence, lack of auxiliary instruments and reagents, immaturity of the reaction system, and high cost. Moreover, the majority of current CRISPR-based methods are unable to address the issues of quantitative detection, multiplex detection, or unbiased interpretation of the results. Until 2020, the first CRISPR commercial product (Sherlock^™^ CRISPR SARS-CoV-2 kit) received FDA emergency use authorization in the U.S. for SARS-CoV-2 detection. Therefore, the rapid translation of CRISPR laboratory results into practice still requires the joint efforts of researchers and enterprises.

## Biosensors and microfluidics

Although only a small number of biosensors and microfluidics are currently available for ASFV detection, they provide vital future directions for ASF diagnostic methods. Microfluidics mainly consists of microchambers, microchannels, or microdroplets, which have the merits of high detection throughput, small reaction volumes, and ease of integration. With the recurrence of the ASF pandemic, several microfluidics-based methods for ASFV detection have been developed.

In the context of ASF circulating across the world, mixed infections caused by different pathogens are currently threatening the pig industry [103]. To develop a rapid, portable and multitarget POCT tool for swine pathogens, Ji et al*.* integrated LAMP into a compact CD-like microfluidic chip for simultaneous detection of five pathogens in portable devices, including gene-deleted and wild-type ASFV, PPV, PRV, PCV2, and PRRSV [104]. This method achieved an LOD of 101 copies/μL for ASFV-MGF505-2R/p72, PPV, and PCV2 and 102 copies/μL for ASFV-CD2v, PRV, and PRRSV. In comparison, our group combined the Hive-Chip with direct LAMP (without DNA extraction) to develop a multiplex and visual detection method that can simultaneously monitor five ASFV genes (B646L, B962L, C717R, D1133L, and G1340L) and largely avoid false negatives by virtue of mutations in a single ASFV gene [7]. Because of high variability and complex genetic composition, on-chip LAMP revealed that the LODs of ASFV synthetic DNA and mock samples were 30 copies/μL and 50 copies/μL, respectively. To evaluate the virus load and virulence, the quantification of ASFV DNA is also necessary. Jia et al*.* utilized nanofluidic chip digital PCR (cdPCR) to detect ASFV at an LOD of 30 1995 copies/reaction, whereas other swine viruses showed no nonspecific amplification [105].

In addition to microfluidics, other technologies, such as graphene, SPR, and nanoplasmonic sensors [106, 107], are also utilized for the development of ASF biosensors. Graphene has the advantages of good conductivity, high carrier mobility, and bipolar electric field effects, so minor charge disturbances on the surface could result in significant changes in the electrical signals of graphene field effect transistors (GFETs). Wang et al*.* presented a cascade detection method based on the Cas12a system coupled with GFET sensors [108]. This method could detect ASFV at concentrations as low as 0.5 aM within 30 min and discriminate the wild-type, CD2V and MGF strains in a single reaction. Another new detection technology, photoelectrochemical (PEC) sensing, relies on the photoelectric conversion signal of the photoelectric active substance to realize the quantitative analysis of the target. Yuan et al*.* reported a PEC biosensor based on Bi/(BiO)2CO3 nanocomposites and SPR for ASFV detection [109]. Combining the high efficiency of in situ LAMP with the excellent performance of PEC, this method has an LOD of 0.048 copies/μL.

In brief, biosensors and microfluidics have great potential for high-throughput, multiplex, portable, and onsite detection of ASFV. The key technical challenge is to integrate on-chip sample preparation, amplification and detection into an all-in-one system [110]. Although many researchers have made great efforts, the current methods are still limited by the high cost of fabrication and production, immature core technologies, difficulty in integration with existing devices, poor compatibility of the materials with biomolecules, and lack of multidisciplinary talent. We believe that with advancements in material science and manufacturing technology, an increasing number of biosensors and microfluidics will be transformed into commercial products in the future.

## The influence of ASFV variants and the injected live attenuated vaccines

In recent years, newly emerging ASFV mutant strains with insertions, deletions, or mutations in the genome have made early diagnosis of ASF more difficult [109]. In 2015, Gallardo et al. reported that the PCR primers recommended by OIE caused false negative results when real samples were detected because of nucleotide mismatches between the primers and the ASFV target gene [111]. In 2019, Bao et al. compared the genomes of the ASFV China/2018/AnhuiXCGQ strain and several related European genotype II strains and reported that 54–107 variant sites contribute to the alteration of amino acid residues in 10–38 genes, which poses a high requirement for accurate detection [17]. Various accessible mutations affect the evolution of ASFV through immune evasion. For efficient viral infection, complex strategies have been developed for ASFV to evade antiviral innate immune responses. For example, ASFV EP364R and C129R block cellular cyclic 2', 3'-cGAMP-mediated antiviral responses for immune evasion [112], and ASFV MGF360‑11L negatively regulates the cGAS‑STING‑mediated inhibition of type I interferon production [113]. More importantly, ASFV variants influence detection accuracy and cause false positives or negatives because of the injected vaccines and mutations in key detection targets.

Mutations related to high virulence have been applied to develop new vaccines and treatments. Hemmink et al. investigated the effects of deleting the CD2v gene from the ASFV-Kenya-IX-1033 strain (ASFV-Kenya-IX-1033-∆CD2v), a genotype IX virus from East Africa. This deletion resulted in reduced virulence and increased overall survival in inoculated pigs, although they presented symptoms such as fever, reduced appetite, and lethargy. Furthermore, 87.5% of these pigs survived a subsequent challenge with the pathogenic parental strain, indicating partial protection [33]. Li et al*.* showed that knocking out the QP509L and QP383R genes from the highly virulent ASFV CN/GS/2018 strain could result in complete viral attenuation in swine [34]. As detected by enzyme-linked immunosorbent assay (ELISA), ASFV-ΔQP509L/QP383R-infected pigs presented low levels of interferon-β (IFN-β), suggesting that deletion of the QP509L/383R gene could reduce ASFV virulence and is promising for vaccine development Similarly, the A137R mutation is another potential choice for developing vaccines. Considering the significant effects of A137R on the attenuation of ASFV virulence in swine, the recombinant virus ASFV-G-ΔA137R was constructed to assess virulence. Animals inoculated intramuscularly with ASFV-G-ΔA137R remained clinically healthy during the 28-day observation period and exhibited a strong virus-specific antibody response, demonstrating that ASFV-G-ΔA137R is a novel live attenuated vaccine candidate [35].

**Figure 3:**
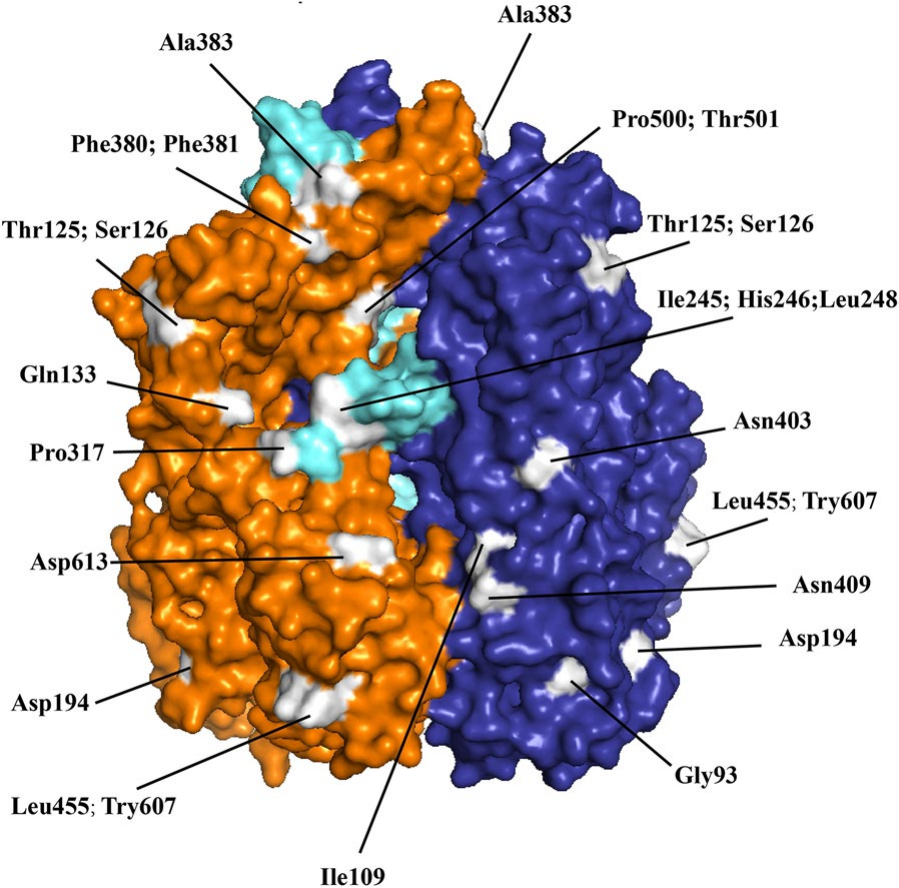
**Locations of all mutations on the surface of p72 encoded by the B646L gene.** The p72 protein structure (pdb:6KU9) was visualized with three subunits, represented by orange, purple, and light green. All p72 mutations were identified via a BLAST search against the entire length of p72 on NCBI. These mutations are visually depicted in white.

However, the emergence of live attenuated vaccines has led to many false positive results. After vaccination, pigs also exhibit symptoms similar to those of ASFV infections, and nucleic acid remains in the body for a period of time [114, 115]. At this point, the commonly used B646L gene can be detected in vaccinated pigs, which easily leads to false-positive results. To distinguish live attenuated vaccines from wild-type ASFV, Zhu et al*.* developed a duplex droplet digital PCR (ddPCR) method based on the B646L and EP402R genes to distinguish EP402R-deleted ASFV strains from wild-type strains [116]. Using MGF505 and p72, Ji et al*.* also reported a rapid and sensitive microfluidic-LAMP chip detection system to discriminate between gene-deleted and wild-type ASFV and four other swine pathogens [104]. In addition to vaccines, evolutionary mutations in the B646L gene are the major source of false negative results. As shown in Figure 3, all the p72 mutations encoded by the B646L gene are marked on the 3D structure. If the mutations are located exactly within the region targeted by the B646L primers, the efficiency and accuracy of detection would largely decrease. To reduce the false-negative results caused by mutations in a single target gene, Zhu et al*.* adopted a multigene detection strategy to establish a visual detection platform for monitoring five target genes and ensuring detection accuracy [7].

Opportunities and challenges coexist. Some emerging mutations are also new directions for ASFV detection. Therefore, we comprehensively summarized the reported genes detected from the perspective of B646L (Table 1) and the other genes (Table 2). Moreover, ASFV gene mutations associated with candidate live attenuated vaccines or naturally occurring lower virulence viruses are summarized in Table 3. We hope to provide valuable and potential objects of study for ASF researchers. For example, the B646L and MGF505-2R genes were chosen by Guo et al*.* for successful construction of a duplex real-time PCR for ASF diagnosis [117]. Another two genes, E111R and I267L, are highly conserved across different genotypes of ASFV, and the latter has been analysed and identified as an early-expressed gene, which is a promising detection target in the future [118, 119]. In brief, high-throughput and multiplex detection constitute a long-term research direction for ASFV infection. The continuous emergence of new variants and the coexistence of multiple swine pathogens are the best proof.

**Table 1:** **Detection methods targeting the B646L gene of ASFV**

Method	Classifications	Strain	Ref.
Based on PCR	A duplex fluorescent quantitative PCR assay	Genotype I and II strains	[132]
	A fast quantitative real-time PCR	ASFV	[46]
	A novel quantitative real‐time PCR	ASFV-China	[45]
	Combination of Fe(OH)_3_ modified diatomaceous earth and qPCR	ASFV in water	[47]
	Nanofluidic chip digital PCR	ASFV	[105]
Based on isothermal amplification	Recombinase-based isothermal amplification assay (RPA/RAA)	Different genotypes ASFV	[70]
	Exonuclease III-assisted recombinase-aided amplification colorimetric assay	ASFV	[72]
	Semiquantitative colorimetric LAMP method	ASFV and HSV	[52]
	A LAMP PCR method	ASFV and gene deleted ASFV	[59]
	RAA nucleic acid test strip assay and double RAA gel electrophoresis detection	ASFV and CSFV	[80]
Based on CRISPR	A CRISPR/Cas12a-SERS platform	ASFV	[95]
	CRISPR/Cas12a combined with immunochromatographic strips	ASFV	[133]
	Cas12a-based portable paper diagnostics	ASFV	[89]
	CRISPR Cas13a-based lateral flow strip	ASFV	[87]
	DNA endonuclease-targeted CRISPR transreporter assay	ASFV	[81]
	CRISPR-Cas12a and fluorescence-based point-of-care system	ASFV	[99]
	Harnessing multiplex crRNAs in the CRISPR/Cas12a system	ASFV	[101]
	CRISPR-Cas12a	ASFV	[93]
	Short palindromic repeats/Cas9-mediated lateral flow nucleic acid assay	ASFV	[96]
	Cas12a-based on-site detection	ASFV	[82]
	CRISPR-Cas12a combined with G-quadruplex	ASFV	[98]
Combination of CRISPR and isothermal amplification	LAMP assay coupled with CRISPR/Cas12a system	ASFV	[90]
	Visual isothermal amplification on trimeric G-quadruplex cis-cleavage activity of Cas12a	ASFV	[97]
	CRISPR-Cas12a combined with LAMP or RPA	ASFV	[91]
	CRISPR/Cas12a-mediated isothermal amplification	ASFV	[65]
	RPA and Cas12a and Cas13a assay	ASFV	[100]

**Table 2:** **Detection methods targeting genes of ASFV other than B646L**

Mutation	Detection method	Strain	Ref.
p54	A novel real-time PCR assay	ASFV	[134]
E248R	A quantitative PCR method	ASFV	[135]
B646L and B438L	A probe-based duplex real-time PCR assay	ASFV	[136]
p72, CD2v and MGF	CRISPR-Cas12a	ASFV	[108]
A137R	Real-time PCR	ASFV	[137]
MGF-360-12L, I177L	Real-time PCR Assays	Highly virulent Georgia strain of ASFV	[138]
E296R	A duplex real-time PCR assay	Genotypes I and II ASFV	[139]
I73R and I329L	The tandem repeat sequences	p72 genotype II and intergenic region (IGR) II variant	[140]
I73R, I329L, O174L, K145R, I329L, I215L	Sequencing and PCR	24 different European II-ASFV genotypes	[111]
B646L, F1055L, and E183L	A triplex real-time quantitative PCR	Genotypes I and II ASFV	[48]
B646L and E183L	A dual real-time PCR Assay	Genotypes I and II ASFV	[141]
E296R	A duplex real-time PCR assay	Genotypes I and II ASFV	[139]
B646L and MGF505-2R	A duplex TaqMan real-time PCR assay	Genotype II ASFV and MGF505-2R gene-deleted	[117]
B646L, EGFP, and mCherry	A triplex PCR Method	Gene-deleted ASFV	[142]
MGF505-2R, EP402R and I177L	A multiplex real-time quantitative PCR assay	Gene-deleted ASFV	[143]
B646L, MGF505-2R and I177L	A triplex crystal digital PCR	ASFV and the MGF505-2R and I177L Gene-Deleted ASFV	[144]
B646L, MGF_360-14L and CD2v	A triplex real-time quantitative PCR	Gene-deleted and wild-type ASFV	[145]
B646L and EP402R	Amplification assays	Gene-deleted ASFV and wild-type ASFV	[70]
B646L, CSFV 5′ UR, PRRSV ORF7 gene	A multiplex qRT-PCR	ASFV, CSFV, PRRSV	[49]
p72 and Cap protein	CRISPR-Cas12a combined with G-quadruplex	ASFV and PCV2	[98]

**Table 3:** **Mutations in candidate live attenuated vaccines**

Mutation	Characteristic	Function	Ref.
F317L	An inhibitor of the NF-kB pathway and production of proinflammatory cytokines	Immune evasion; promote viral replication	[146]
EP364R, C129R	Target cyclic GMPAMP to inhibit the cGAS-STING signalling Pathway	Immune evasion; promote viral replication	[112]
MGF360-9L, MGF505-7R	Decrease ASFV-specific IFN-g response and increase macrophage infiltration	Promote viral replication; enhance virulence	[147]
H108R	/	Promote viral replication; enhance virulence	[148]
A137R	/	Promote viral replication; enhance virulence	[35]
E165R	Encode dUTPase	Promote viral replication	[149]
E184L	A transcribed gene in the infectious cycle	Enhance virulence	[150]
DP148R, DP71L, DP96R	/	Immune evasion; enhance virulence	[151]
L7L-L11L	Clustered gene on open reading frames and located at the right variable region	Enhance virulence	[152]
I177L	Belong to ASFV-G genome	Enhance virulence	[153]
EP402R	Hemagglutinin of ASFV	Immune evasion; enhance virulence	[154]

## Disease dynamics and optional detection methods for ASF

**Figure 4:**
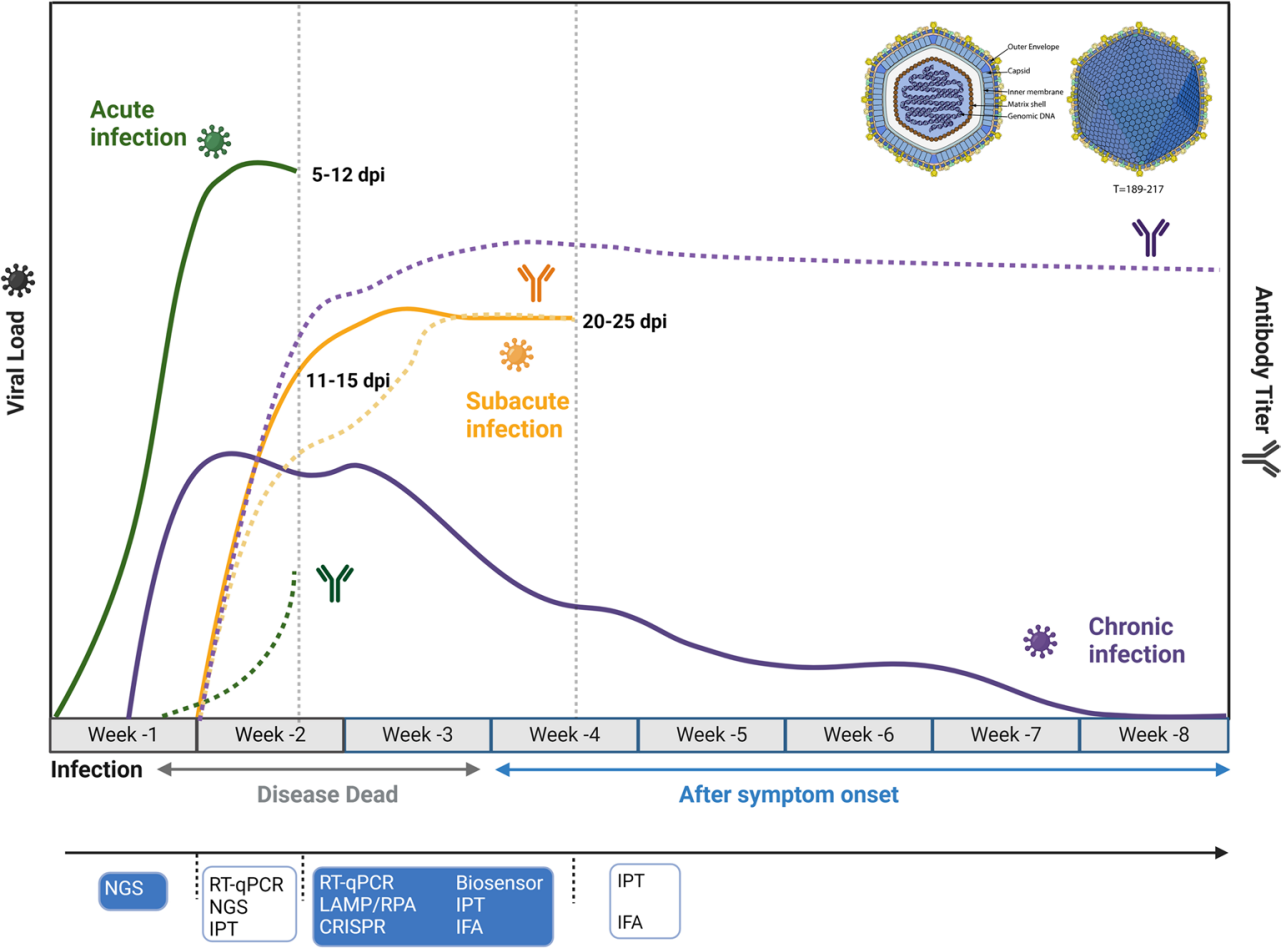
**Disease dynamics and recommended detection methods for ASFV.** ASFV infections are typically divided into three types, namely, acute, subacute, and chronic, which involve different changes in the viral load and antibody expression over a period of time. It is necessary to utilize different detection strategies at different phases. The solid and dashed lines indicate changes in the viral load and antibody titre, respectively. The green, orange and purple colours indicate acute, subacute, and chronic infections, respectively.

According to their clinical symptoms, ASFV infections can generally be classified as acute, subacute or chronic. In acute infection, pigs usually die within 5–7 days, and some pigs can even die abruptly without any signs [120]. Various symptoms, including high fever (40 ~ 42 ℃), anorexia, lethargy, and subdermal hemorrhages, accompanied by high replication of DNA and low antibody titres, are manifested in infected pigs [121]. In subacute infection, pigs exhibit less severe symptoms, such as lower fever (~40.5 °C), tachypnea, and diarrhea [122]. Pigs can survive days to weeks, accompanied by an increased viral load and serum antibody titre, and some of them can progress to chronic infection. Unfortunately, owing to the durable virulence of ASFV, pigs with chronic infection cannot recover from difficulty breathing and delayed growth [123]. Typically, pigs with chronic infection can survive several months, but recovery is difficult. As shown in Figure 4, high expression of ASFV-induced antibodies results in a far longer duration than that of the viral load, despite the earlier increase in the viral load.

Considering the different types and phases of ASFV infections, we can utilize different detection methods. Owing to the low viral load within 7 days post infection (dpi), NGS can achieve real-time surveillance with high sensitivity and accuracy, and it is suitable for rapidly identifying unknown pathogens and new evolutionary mutations [124–126]. However, NGS is usually limited by its high cost, specialized operators, and long analysis time, making it unsuitable for clinical application at a large scale. With an increased viral load within 7–10 dpi, RT‒qPCR and indirect immunoperoxidase test (IPT) were performed prior to NGS for the detection of acute and chronic ASFV infection. Notably, viral replication is slightly slower in pigs with subacute infection, indicating the importance of NGS for ASFV detection. During 11–25 dpi in subacute and chronic infections, nucleic acid detection methods, including RT‒qPCR, LAMP, CRISPR and biosensors, as well as antibody detection methods, including IPT and indirect immunofluorescence assay (IFA), can be applied because of the high level of DNA replication and antibodies. With the prolongation of ASFV infection, antibody detection is gradually replacing nucleic acid detection as a more reliable method [127]. In conclusion, a better understanding of the benefits and drawbacks of different methods and the clinical characteristics of ASFV can provide new perspectives for the detection of ASFV.

## Discussion

Since its initial identification in the early twentieth century, African swine fever virus (ASFV) has been a persistent global concern, as it has circulated across various regions without the support of effective vaccines or treatments. This situation underscores the critical need for accurate and timely diagnosis of ASFV infections. In this comprehensive review, we delve into an array of nucleic acid detection methods for ASFV, including sequencing, PCR, isothermal amplification, CRISPR, biosensors, and microfluidics, which offer detailed evaluations of each method. Given the complexities of the ASF virus load and antibody dynamics, it is imperative to employ tailored detection strategies at different stages of infection, highlighting the importance of field-deployable diagnostics in ASF prevention and control.

False positives and negatives present significant challenges in ASF diagnosis, with evolutionary mutations in the B646L gene being a common source of false negatives—particularly mutations affecting primers and probe target regions. Conversely, the use of live attenuated vaccines (e.g., EP402R-deleted vaccine candidates) often leads to false positives [116]. Factors such as the integrity of the collected samples, the effectiveness of sample processing, and the cleanliness and organization of the workspace can significantly impact detection outcomes, potentially leading to inaccurate results. Minimizing aerosol contamination is crucial, as the dynamics of the virus load and antibody titres may surpass the detection limits of certain methods, necessitating the selection of appropriate diagnostic tools.

We anticipate two primary research directions for ASF diagnosis. First, sample preparation remains a bottleneck for fully integrated detection methods, particularly for POCT devices. Despite the use of various DNA extraction techniques, separating sample treatment from detection platforms complicates the process and usage of the platform. A simplified, direct-from-sample-to-answer process is urgently needed to increase the detection efficiency of ASFV. While some researchers have developed sample preparation-free methods using PCR and LAMP, there remains a significant scope for improving the stability, sensitivity, and compatibility of these methods.

Second, the growing volume of samples and variants challenges the capacity of conventional PCR and isothermal amplification methods to meet the demands for throughput, multiplicity, and portability. Although the Biofire Company launched the FilmArray 2.0 platform, the design actually stacks multiple FilmArray 1.0 devices together to improve throughput, and each device is expensive and not portable [128, 129]. Recently, Zhu et al*.* introduced HiCube, a breakthrough platform that combines the Hive-Chip with LAMP [130]. This iPad-controlled, high-throughput, multiplex, and portable detection system offers an ideal solution for managing a high volume of samples with enhanced multiplicity and portability. The integration of sample treatment into HiCube could position this platform as a leading platform for monitoring animal and zoonotic epidemics. Moreover, biosensors or microfluidics, which isolate different primers in microchambers or microchannels, offer a promising avenue for multiplex detection in a contained environment.

In conclusion, the development of novel POCT biosensors for ASFV detection—including electrochemical, magnetic, field-effect transistor (FET)-based, and enzyme-based sensors—holds significant promise. These advancements could enable pig farmers to quickly identify ASF and respond promptly, reducing reliance on central laboratories. As we move forward, the emergence of practical POCT methods for diagnosing ASF and other infectious diseases is anticipated, heralding a new era in disease management and control.
